# Accidental Ingestion of Hearing Aid with Exposed Battery

**DOI:** 10.5811/cpcem.2017.1.33281

**Published:** 2017-03-16

**Authors:** Ethan B. Kunstadt, Mark I. Langdorf

**Affiliations:** University of California, Irvine, Department of Emergency Medicine, Irvine, California

## Abstract

A 59-year-old female presented to the emergency department (ED) three days after accidental ingestion of an intact in-the-ear hearing aid. This is the first report of ingestion of a complete hearing aid traveling past the gastroesophageal junction. Of concern was the exposed battery attached to the hearing aid that had advanced minimally in the three days since last evaluation. This case report discusses her ED testing, including gastroenterology consultation, and ultimately retrieval from her distal stomach. The authors conclude that this removal was not medically necessary.

## INTRODUCTION

Hearing loss occurs in some 30% of adults greater than 60, and has been associated with increased risk of dementia and falls.^1^, ^2^ Given the stigma that may arise from hearing loss, advances in design of hearing devices have taken us from conspicuous wearable metal “ears” and ear trumpets in the 17^th^ and 18^th^ centuries to the present-day technologies. Cochlear implants enhance sound transmission to the vestibulocochlear organ, and miniaturized within-ear devices amplify and compensate for conductive and sensorineural hearing loss.

In-the ear (ITE), in-the-canal, and completely-in-canal hearing aids are devices small enough to cause risk of ingestion in the elderly or cognitively impaired. Foreign body ingestion is a relatively common chief complaint in the emergency department (ED), but most cases occur in children with peak incidence between six months and six years of age. Eighty percent of foreign bodies pass spontaneously through the gastrointestinal tract; surgical intervention is required in only 12–16%. Death is extremely rare. One study reported no deaths among 852 adults, while another in children reported one out of 2,206.^3^ Impaction, perforation, or other complications tend to occur at areas of gastrointestinal (GI) narrowing or angulation; however, once the object has passed the esophagus, almost all foreign bodies that are not sharp pass uneventfully.

We present the first reported case of an adult accidently ingesting a complete hearing aid with exposed battery.

## CASE REPORT

A 59-year-old Caucasian female presented to the ED with a chief complaint of accidentally swallowing her hearing aid. The patient denied symptoms aside from anxious thoughts regarding the ingestion. She swallowed the device accidentally three days before, when she grabbed a handful of acetaminophen from her night stand, which she takes each morning for osteoarthritis. The patient went to work on the day of the ingestion, but left early given mild discomfort in her chest and concern about the swallowed hearing aid. An abdominal radiograph done at an outside hospital revealed the foreign body at the gastroesophageal (GE) junction. The patient was sent home and instructed to carefully examine her stool for passage of the foreign body.

**Image 1:**
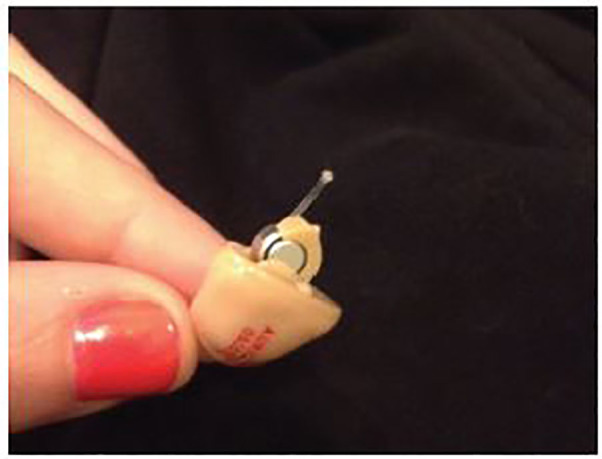
An in-the-ear hearing aid similar to our patient’s, in the off position with battery exposed.

On presentation to our ED, the patient’s chest discomfort was resolved; however, she was concerned because she had not seen the foreign body in any of her stools. She had taken polyethylene glycol 3350 (Miralax) daily in hopes this would promote passage. She denied shortness of breath, cough, dysphagia or odynophagia, abdominal pain, nausea, vomiting, or changes in bowel habits. The patient brought her matching hearing aid, which was not ingested: an ITE device, approximately 2cm in diameter with plastic casing. However, when the hearing aid is in the off position, as it was when it was ingested, a small battery protrudes from the device and is not protected within plastic casing (Image 1).

The patient had osteoarthritis, depression, and attention deficit hyperactivity disorder, with two caesarean sections, and she denied tobacco, alcohol, or illicit drugs.

On physical exam, the patient was well-appearing, in no apparent distress and was breathing comfortably. Initial vital signs were temperature 36.6C, blood pressure 153/93 mmHg, heart rate 106 beats per minute, respiratory rate 16 breaths per minute, and O_2_ saturation 100% on room air. Tachycardia was resolved by the time of exam by the physician. Oropharynx revealed moist oral mucosa, no pharyngeal erythema, exudate or fullness, and uvula was midline. There was no reproducible chest wall discomfort or subcutaneous crepitus of the chest. Breathing was non-labored with no accessory muscle use. Breath sounds were clear and equal bilaterally with no wheezes or rhonchi. Cardiac auscultation was normal. Radial pulses were normal and equal. Her abdomen was soft, non-distended, and non-tender, with normal bowel sounds.

**Image 2:**
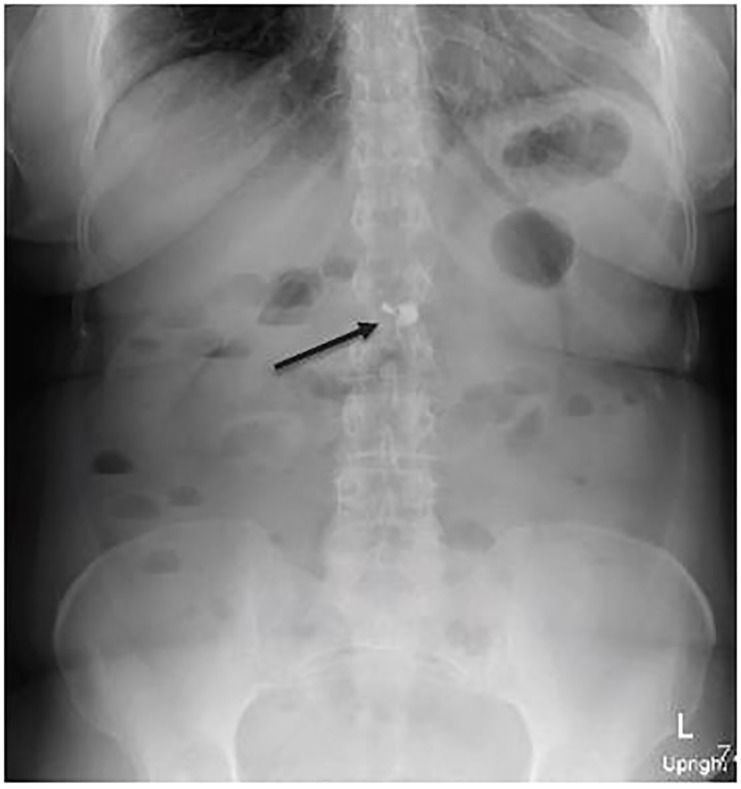
Anterior-posterior upright film with foreign body visualized in mid-upper abdomen (arrow).

An abdominal radiograph was done to localize the foreign body, as the patient reported the hearing aid was easily identifiable in the distal esophagus on plain films three days prior (Image 2).

Final read of the plain films was: “3 closely grouped metallic densities measuring 2 mm, 6 mm and 12 mm in size project over the midline upper abdomen at the level of L1–2. Findings may represent the hearing aid/foreign body of interest.”

**Image 3:**
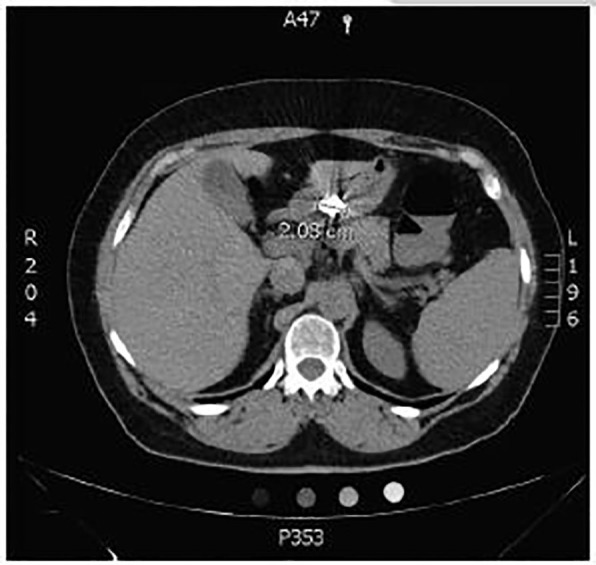
Axial view from computed tomography of abdomen and pelvis showing a 2cm foreign body in the stomach.

**Image 4:**
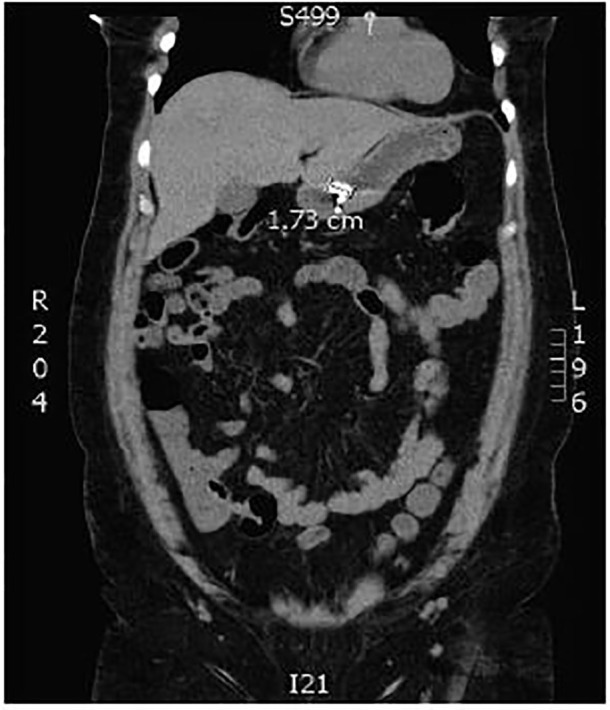
Coronal view from computed tomography of the abdomen and pelvis noting the foreign body in the distal stomach.

Emergency physicians (EP) consulted the gastroenterology service after the radiographs were shot; however, neither team could determine the precise location of the foreign body from these films alone. Non-contrast computed tomography (CT) of the abdomen and pelvis was then obtained per GI’s request for further evaluation of the foreign body’s size and location (Images 3 and 4).

While awaiting CT results, EPs spoke with the American Association of Poison Control Centers (1-800-222-1222) regarding this case. Their representative reassured us that exposed batteries are only a concern if they are still in the esophagus, due to constant contact with the esophageal mucosa, which allows for injury of the mucosa. Assuming the hearing aid was at least beyond the esophagus by that point, the patient was expected to safely pass the foreign body in the coming days.

The final radiology report concluded that the foreign body was in the mid-upper abdomen, but could not confidently state the exact location.

The GI team determined the object appeared to be in the distal gastric body/antrum. After examining the patient’s other hearing aid, and the size of the object on CT, they felt the object would eventually pass uneventfully, even if the exposed battery were to entirely separate from the hearing aid. However, as the patient continued to express concern, she was offered an esophagogastroduodenoscopy.

The patient had a successful endoscopy under general anesthesia the following morning with retrieval. The hearing aid was removed intact with battery still in place using a Roth net. There was no evidence of esophagitis, erosions, or ulcerations. The patient tolerated the endoscopy well with only a mild sore throat.

## DISCUSSION

This is the first report of ingestion of an ITE hearing aid traveling past the gastroesophageal junction. We could find only one other case of an 86-year-old man accidentally ingesting a whole hearing aid, but this was a larger behind-the-ear (BTE) device.^4^ He presented with acute dysphagia, and his device was discovered in the hypopharynx. The object was uneventfully removed once the connecting tube was disconnected from the coupling device, which had lodged in the upper esophageal sphincter. The remaining molded inner-ear hearing aid portion remained distal to the BTE portion in the proximal esophagus. This remaining portion was able to be removed with endoscopy and the patient was able to swallow immediately following the procedure.

## CONCLUSION

While foreign body ingestions are common, this case is unique because a potentially dangerous exposed battery remained in the stomach for three days with minimal advancement. Exposure of stomach mucosa to the battery with potential for impaction proximal to the pylorus is a unique situation. Consensus between EPs, GI and poison center determined the situation to be non-emergent. Although comforting to the patient, we believe that endoscopic removal of the hearing aid, despite the exposed battery, was unnecessary and it would have passed given sufficient time.
